# Papers on Genito-Urinary Surgery

**Published:** 1919-01

**Authors:** 


### Important Papers.

In this issue of the Texas Medical Journal will appear the first of a series of articles by Dr. Gustav Kolischer of Chicago, Illinois.

If one reads this first essay on Hypertrophy of the Prostate Gland, he may then surmise the completeness of the series of articles which will include Technique of Suprapubic Prostatectomy, Tumors Of The Urinary Bladder, The Pus Kidney, and the Technique of Nephrotomy and Nephrectomy.

In this first article one can see that the author has given no little study to the subject and from the number of characteristics mentioned, which are not commonly brought out in the discussion of this subject, it also appears that he has had a very extensive amount of clinical experience. One interested in Genito-Urinary surgery will do well to preserve these papers for, when complete, the series will be very interesting and valuable.

